# Simultaneous optical flow and source estimation: Space–time discretization and preconditioning

**DOI:** 10.1016/j.apnum.2015.04.007

**Published:** 2015-10

**Authors:** R. Andreev, O. Scherzer, W. Zulehner

**Affiliations:** a RICAM, Austrian Academy of Sciences, Altenberger Str. 69, 4040 Linz, Austria; b Computational Science Center, University of Vienna, Oskar-Morgenstern Platz 1, 1090 Wien, Austria; c Institute of Computational Mathematics, Johannes Kepler University, Altenberger Str. 69, 4040 Linz, Austria

**Keywords:** Optical flow, Space–time, Discretization, Preconditioning, Saddle-point, Linear-quadratic control

## Abstract

We consider the simultaneous estimation of an optical flow field and an illumination source term in a movie sequence. The particular optical flow equation is obtained by assuming that the image intensity is a conserved quantity up to possible sources and sinks which represent varying illumination. We formulate this problem as an energy minimization problem and propose a space–time simultaneous discretization for the optimality system in saddle-point form. We investigate a preconditioning strategy that renders the discrete system well-conditioned uniformly in the discretization resolution. Numerical experiments complement the theory.

## 1 Introduction

Optical flow is the apparent motion of objects, surfaces, and edges in a visual scene. In Computer Vision the optical flow is calculated as the flow field registering pixels of a movie, which we understand as a sequence of ordered images. The classical optical flow equation is based on the following assumptions:

1. **Brightness constancy** along each characteristic of the flow.
2. A **slowly varying** image sequence.

The first assumption implies that changes of intensities caused by varying illumination (for instance shades) influence and distort the recovered flow. In this paper we admit violations of this assumption, and aim at estimating global changes of illumination and the effect of them onto the flow field. Indeed, the problem we consider here consists in the simultaneous determination of the optical flow, the brightness, *and* its possible sources and sinks in space and time. While standard optical flow algorithms process two successive frames of a movie sequence at a time [18–20,3,13,6,1], we work with a continuous space–time simultaneous formulation similarly to [23]. A brief overview of gradient based optical flow models and algorithms may be found in [14].

Related to our work is [24]. Therein, the standard optical flow constraint was replaced by the continuity equation $\partial_{t}\rho +\nabla \cdot (\rho v)=0$, motivated by mass conservation in fluid flow. We relax the continuity equation by allowing distributed *sources g*, and replace the momentum *ρv* by one unknown *j*, called the *flux*; we thus obtain the constraint $\partial_{t}\rho +\nabla \cdot j=g$.

The work [17], see also references therein, suggested incorporating physical models to account for illumination changes, such as the diffusion of the heat observed by infrared cameras. The parameters governing the physical model are then estimated in parallel with the optical flow. Our ansatz is more naive, in the sense that we treat the possible change in illumination as an additional unknown, but a physical description can in principle be used instead.

As a further difference to the bulk of optical flow literature we treat the density *ρ* as an unknown. We thus estimate (ρ,j,g) in parallel by minimizing a functional J which consists of a discrepancy term for *ρ* and regularization terms for *j* and *g*. This is similar to [7]; however, using the flux *j* instead of the velocity *v* results in *linear* optimality conditions characterizing the minimizer of the functional J. While this is convenient for computation, it leads to somewhat unexpected flow patterns (see Sections 5–6).

It is important to note that these optimality conditions form a set of equations that are coupled in space–time. This is typical for optimal control problems with spatio-temporal constraints due to the coupling of the original problem forward in time and the adjoint problem backward in time. The main contribution of this work is to propose and test a preconditioner for the discretized space–time system in saddle-point form that is robust in the discretization parameters.

The outline of the paper is as follows. In Section 2 we introduce our optical flow model, which consists in the minimization of a quadratic cost functional over a space of space–time dependent functions subject to an optical flow constraint. In Section 3 the functional analytic framework is established. In Section 4 we rewrite the optimality conditions in a saddle-point form and discuss its discretization and preconditioning. In Section 5 we report on our numerical experiments. Section 6 concludes the paper.

In the following, 〈⋅,⋅〉 denotes the duality pairing, while ${\langle \cdot ,\cdot \rangle }_{X}$ and ${\left\|\cdot \right\|}_{X}$ denote the scalar product and the norm of a Hilbert space *X*. The usual Lebesgue and Sobolev spaces on a domain *D* are written as $L_{p}(D)$ and $H^{s}(D)$. We abbreviate ${\left\|\cdot \right\|}_{L_{2}(D)}$ as ${\left\|\cdot \right\|}_{D}$, and similarly for other $L_{2}$ spaces.

## 2 Optical flow model

We consider a movie sequence of time length T>0, with each frame defined on a rectangular domain $D\subset R^{2}$. We write J:=(0,T) for the temporal interval. As explained in the introduction above, our optical flow model is the scalar transport equation(1)
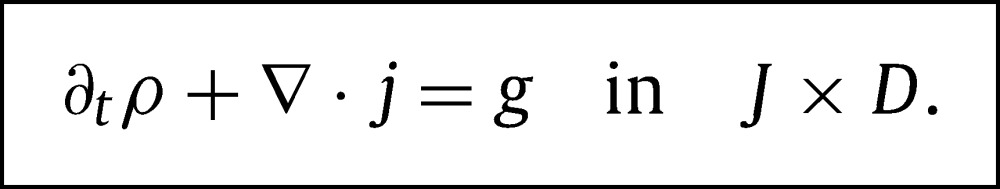
 Here, *ρ* denotes the *density* which is the intensity of the image considered as a piecewise constant function; *j* denotes the *flux* which is the optical flow weighted by the density; and *g* is a function that models varying spatial illumination. The divergence ∇⋅ acts on the spatial variable only. The functions *ρ* and *g* are scalar-valued, while *j* is vector-valued on $\overset{¯}{J}\times D$. Integrating (1) over a subdomain $D^{\prime }\subset D$ and using the divergence theorem for the *j* term, one sees that the density *ρ* is either transported in or out of $D^{\prime }$ over its boundary, or is created/annihilated by means of the source term *g*.

In this paper we consider the problem of identifying the flux *j and* the source *g* from a finite number of frames of a movie, indexed by $T\subseteq \overset{¯}{J}$:

(2) $$\rho_{\tau }\in L_{2}(D),\;\tau \in T.$$

We aim at minimizing the data fidelity functional(3) subject to the transport equation (1) and further constraints on the flux *j* and the source *g* as discussed below. Here, the symbol  denotes the averaged sum $\frac{1}{\#T}\sum_{\tau \in T}$.

For comparison purposes we recall the standard optical flow equation, which reads as follows:

(4) $$\partial_{t}\rho +\Phi \cdot \nabla \rho =0\;\text{in}\;J\times D.$$

Eq. (4) can be formally derived from (1) by identifying *j* and *ρ*Φ and neglecting small terms: Indeed, from (1) we get

(5) $$\partial_{t}\rho +\Phi \cdot \nabla \rho +\rho \nabla \cdot \Phi =0\;\text{in}\;J\times D.$$

Hence, if the term ρ∇⋅Φ is small (meaning that there is a slowly varying velocity), and if there are no changes in illumination, the equations are identical.

## 3 Functional analytic framework

We now introduce function spaces for which the transport equation (1) is well-defined. We shall work with image intensity $\rho \in H^{1,0}:=H^{1}(J;L_{2}(D))$, flux $j\in H^{0,\mathrm{div}}:=L_{2}(J;H^{\mathrm{div}}(D))$, and source $g\in H^{0,0}:=L_{2}(J;L_{2}(D))$. The superscripts indicate the Sobolev smoothness in time and space, respectively. Here, $H^{\mathrm{div}}(D)$ is the space of vector-valued functions in ${\left[L_{2}(D)\right]}^{2}$ with distributional divergence in $L_{2}(D)$. As a consequence of the Fubini–Tonelli theorem [25, Section 0.3], the space $H^{0,0}$ is isometrically isomorphic to $L_{2}(J\times D)$. The norms on $H^{1}(J)$ and $H^{\mathrm{div}}(D)$ are defined by

$${\left\|f\right\|}_{H^{1}(J)}^{2}:=T^{-2}{\left\|f\right\|}_{J}^{2}+{\left\|f^{\prime }\right\|}_{J}^{2},\;f\in H^{1}(J),$$

$${\left\|v\right\|}_{H^{\mathrm{div}}(D)}^{2}:=\mathrm{diam}\;{\left(D\right)}^{-2}{\left\|v\right\|}_{D}^{2}+{\left\|\nabla \cdot v\right\|}_{D}^{2},\;v\in H^{\mathrm{div}}(D),$$

where the scaling $T^{-2}$ and $\mathrm{diam}\;{\left(D\right)}^{-2}$ is to match the units. We introduce the product space

$$X:=H^{1,0}\times H^{0,\mathrm{div}}\times H^{0,0}$$

and associate it with the norm

(6) $${\left\|(\rho ,j,g)\right\|}_{X}^{2}:={\left\|\rho \right\|}_{H^{1,0}}^{2}+{\left\|j\right\|}_{H^{0,\mathrm{div}}}^{2}+{\left\|g\right\|}_{H^{0,0}}^{2}.$$

The transport equation constraint (1) is reformulated via the linear operator

(7) $$G:X\to H^{0,0},\;G(\rho ,j,g):=\partial_{t}\rho +\nabla \cdot j-g.$$

Since *G* is continuous, the preimages of closed subsets are closed; in particular, its kernel

(8) $$\Gamma :=G^{-1}(0)\subset X$$

is a closed linear subspace. It contains precisely the triples (ρ,j,g)∈X that satisfy the transport equation (1).

Given two regularization parameters $\alpha_{j}>0$ and $\alpha_{g}>0$, we define the penalization functional(9)
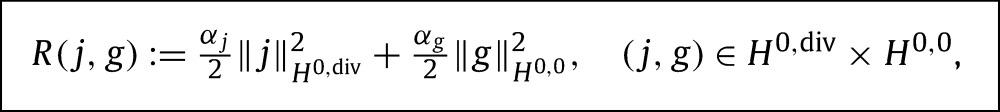
 and, recalling $F_{T}$ from (3), also the cost functional(10)
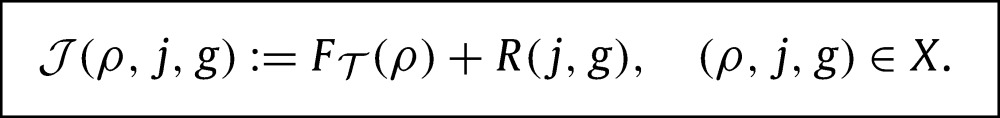
 The parameters $\alpha_{j}$ and $\alpha_{g}$ are dimensionless. The following lemma is straightforward.

Lemma 1 *The functional* J *is Gâteaux differentiable on X. With* $\rho_{\tau }$ *from* (2)*, its Gâteaux derivative at* (ρ,j,g)∈X *is the continuous linear functional* $J^{\prime }(\rho ,j,g)=A(\rho ,j,g)-\ell$*, where* $A:X\to X^{\prime }$ *and* $\ell \in X^{\prime }$ *are given by*(11)(12)
*Moreover, A is continuous, self-adjoint, and* Γ*-elliptic,*

(13) $$\exists \alpha >0:\;\langle Av,v\rangle \geq \alpha {\left\|v\right\|}_{X}^{2}\;\forall v\in \Gamma .$$

*Further, A induces a seminorm* ⦀⋅⦀ *on X given by*(14)
*This seminorm is in fact a norm on* $\Gamma =G^{-1}(0)$*, where it is equivalent to* ${\left\|\cdot \right\|}_{X}$*.*

With the help of this lemma and standard arguments from variational calculus, one can show strict convexity and coercivity properties of the functional J:

Lemma 2 *The functional* J *defined in* (10) *is continuous and convex on X. Moreover, it is strictly convex on* Γ*,*

(15) $$J(\lambda v_{+}+(1-\lambda )v_{-})<\lambda J(v_{+})+(1-\lambda )J(v_{-})\;\forall v_{\pm }\in \Gamma ,\;\lambda \in (0,1),$$

$v_{+}\neq v_{-}$*, and coercive on* Γ*,*

(16) $$\exists \alpha >0,\;\beta \geq 0:\;J(v)\geq \alpha {\left\|v\right\|}_{X}^{2}-\beta \;\forall v\in \Gamma .$$

Our optical flow problem now reads as follows:

Definition 1 For given regularization parameters $\alpha =(\alpha_{j},\alpha_{g})$, the optical flow $u_{\alpha }$ is the unique minimizer of the functional J over Γ.

Existence of the minimizer is due to standard arguments of the calculus of variations [12], because J is non-negative and proper (J≠∞) on Γ. Uniqueness follows from the strict convexity (15) of J on Γ.

The minimizer $u_{\alpha }$ of J is equivalently characterized in terms of first order optimality conditions. By [12, Theorems 1.3–1.4 in §3.1.3], we have $J^{\prime }(u_{\alpha })=0$, where $J^{\prime }(u)\in \Gamma^{\prime }$ is the Gateaux derivative of J at u∈Γ. To simplify the notation we will omit the dependence on *α*. Using Lemma 1, the requirement $J^{\prime }(u)=0\in \Gamma^{\prime }$ is equivalent to the variational problem(17)
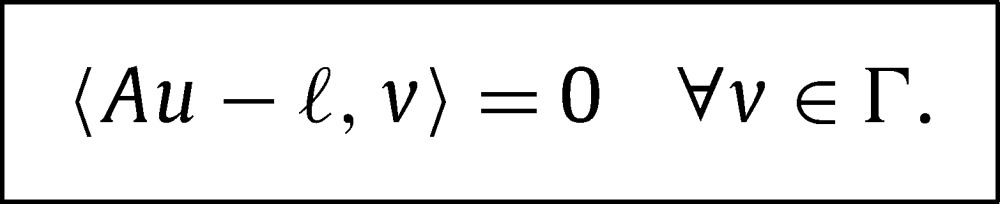
 The fact that this variational problem is posed on the implicitly defined subspace $\Gamma =G^{-1}(0)$ leads to the saddle-point problem introduced in the next section.

## 4 Numerical solution

In order to minimize J we solve the equivalent variational problem (17). The constraint u∈Γ is implemented using a Lagrange multiplier *λ* to complement (17) to a saddle-point problem: Find $(u,\lambda )\in X\times H^{0,0}$ such that

(18) $$\langle Au,v\rangle +\langle Gu,\mu \rangle +\langle Gv,\lambda \rangle =\langle \ell ,v\rangle \;\forall (v,\mu )\in X\times H^{0,0}.$$

### 4.1 Discretization

In order to discretize (18) we introduce finite dimensional subspaces $X_{h}\subset X$ and $M_{h}\subset H^{0,0}$, as specified below, and consider the discrete saddle-point system: Find $(u_{h},\lambda_{h})\in X_{h}\times M_{h}$ such that

(19) $$\langle Au_{h},v\rangle +\langle Gu_{h},\mu \rangle +\langle Gv,\lambda_{h}\rangle =\langle \ell ,v\rangle \;\forall (v,\mu )\in X_{h}\times M_{h}.$$

With $A_{h}:X_{h}\to X_{h}^{\prime }$ and $G_{h}:X_{h}\to M_{h}^{\prime }$, defined by $A_{h}v:=(Av){|}_{X_{h}}$ and $G_{h}v:=(Gv){|}_{M_{h}}$, $v\in X_{h}$, as well as $\ell_{h}:=\ell {|}_{X_{h}}$, the system (19) is equivalent to(20)
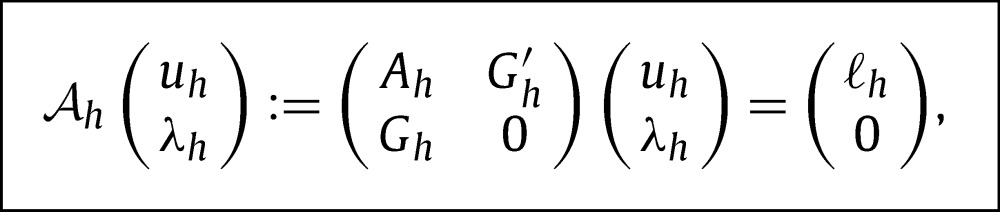
 where $G_{h}^{\prime }:M_{h}\to X_{h}^{\prime }$ is the $H^{0,0}$-adjoint.

Analogously to the continuous case, we set

(21) $$\Gamma_{h}:=\{u_{h}\in X_{h}:\langle Gu_{h},\mu \rangle =0\;\forall \mu \in M_{h}\}.$$

The Brezzi equivalence theorem, see [8, Satz III.4.3], states that the left-hand-side of (19) defines an isomorphism $X_{h}\times M_{h}\to X_{h}^{\prime }\times M_{h}^{\prime }$ if and only if the following two conditions are fulfilled:

1. *A* is $\Gamma_{h}$-elliptic,
  (22) $$\exists \alpha_{h}>0:\;\langle Av,v\rangle \geq \alpha_{h}{\left\|v\right\|}_{X}^{2}\;\forall v\in \Gamma_{h}.$$
2. The discrete inf-sup constant is positive,
  (23) $$\beta_{h}:=\underset{\lambda \in M_{h}\setminus \{0\}}{\inf}\underset{v\in X_{h}\setminus \{0\}}{\sup}\frac{\langle Gv,\lambda \rangle }{{\left\|v\right\|}_{X}{\left\|\lambda \right\|}_{H^{0,0}}}>0.$$

We shall employ a discretization $X_{h}\times M_{h}\subset X\times H^{0,0}$ for which the two rather restrictive conformity conditions

(24) $$\Gamma_{h}\subset \Gamma \;\text{and}\;M_{h}\subset GX_{h}$$

hold. The first, together with Γ-ellipticity (13) of *A*, implies $\Gamma_{h}$-ellipticity (22) of *A*, and the second immediately implies the discrete inf-sup condition (23) with $\beta_{h}\geq 1$.

The square domain *D* is partitioned into non-overlapping open rectangles and/or triangles, collected in $D_{h}$. Geometric compatibility conditions are imposed following [11, p. 51]: each edge of any geometric element $K\in D_{h}$ is either part of the boundary ∂*D* or is also an edge of some other geometric element $K^{\prime }\in D_{h}$. By $D_{h}^{\partial }$ we mean the set of edges *E* of all geometric elements in $D_{h}$ (shared edges occur only once). The temporal interval J=(0,T) is partitioned into open subintervals collected in $J_{h}$, such that $\overset{¯}{J}={⋃}_{I\in J_{h}}\overset{¯}{I}$. Quantities in $H^{0,0}$, such as $\partial_{t}\rho$, ∇⋅j, *g*, and *λ*, are discretized as piecewise constants on each space–time geometric element I×K, $(I,K)\in J_{h}\times D_{h}$. The conserved quantity $\rho \in H^{1,0}$ is discretized by continuous functions $J\to L_{2}(D)$ that are affine on each $I\in J_{h}$ and have values in the space of piecewise constant functions on $D_{h}$. The flux $j\in H^{0,\mathrm{div}}$ is discretized by assigning a flux density $j_{IE}$ to each pair $(I,E)\in J_{h}\times D_{h}^{\partial }$, such that $j_{IE}=\frac{1}{\left|I\|E\right|}\int_{I}\int_{E}j(t,x)\cdot n(x)d\sigma (x)dt$, and interpolated linearly into the inner of each geometric element $I\times K\in J_{h}\times D_{h}$. This is trivially possible on each rectangle, and corresponds to Raviart–Thomas interpolation on triangles [8, p. 141]. It is then clear that the conformity conditions (24), and therefore (22) and (23), are satisfied.

### 4.2 Preconditioning

Several classes of preconditioners have been developed for saddle-point systems of the form (20), see the survey [4]. Preconditioners based on computationally efficient approximations of the inverses of $A_{h}$ and of the Schur complement $S_{h}=G_{h}A_{h}^{-1}G_{h}^{\prime }$ are widely used. However, our $A_{h}$ is not, in general, invertible on the whole finite-dimensional space $X_{h}$. Indeed, recall from (11) that *A* has the block structure(25) where v=(ρ,j,g), so that the first block is not positive definite on $H^{1,0}$.

In [5] a similar saddle-point system for a related problem from image registration (which corresponds to #T=2 in our case) was obtained, for which block-triangular preconditioners were proposed. We replace the triangular preconditioner by the symmetric and indefinite preconditioner $P_{h}:X_{h}\times M_{h}\to X_{h}^{\prime }\times M_{h}^{\prime }$, given by

(26) $$P_{h}:=\left(\begin{array}{cc} {\overset{ˆ}{A}}_{h} & 0\\ G_{h} & I \end{array}\right)\left(\begin{array}{cc} {\overset{ˆ}{A}}_{h}^{-1} & 0\\ 0 & -{\overset{ˆ}{S}}_{h} \end{array}\right)\left(\begin{array}{cc} {\overset{ˆ}{A}}_{h} & G_{h}^{\prime }\\ 0 & I \end{array}\right),$$

see [2]. It was shown in [21] that this allows to transform the indefinite saddle-point system into a symmetric positive definite one for a new inner product (see (27) below), so that the conjugate gradient (CG) method can be applied. This is not possible for the preconditioned system of [5] where the spectrum is nonreal. Another well-known method to transform the indefinite saddle-point system into a symmetric positive definite one was proposed in [10,9]. There it is also required that $A_{h}$ be positive definite on $X_{h}$, which is not true in our case.

It was shown in [21, Theorem 2.1] that if

(27) $$N_{h}:=\left(\begin{array}{cc} {\overset{ˆ}{A}}_{h}-A_{h} & 0\\ 0 & G_{h}{\overset{ˆ}{A}}_{h}^{-1}G_{h}^{\prime }-{\overset{ˆ}{S}}_{h} \end{array}\right)$$

is positive definite on $X_{h}\times M_{h}$ then $P_{h}^{-1}A_{h}$ is symmetric positive definite with respect to the scalar product defined by $N_{h}$. To that end, assume:

1. ${\overset{ˆ}{A}}_{h}>A_{h}$ and $G_{h}{\overset{ˆ}{A}}_{h}^{-1}G_{h}^{\prime }>{\overset{ˆ}{S}}_{h}$ to assert that $N_{h}>0$, as well as
2. ${\overset{ˆ}{A}}_{h}\leq M_{0}A_{h}$ on $\ker G_{h}$, for some real constant $M_{0}\geq 1$,
3. $G_{h}{\overset{ˆ}{A}}_{h}^{-1}G_{h}^{\prime }\leq M_{1}{\overset{ˆ}{S}}_{h}$ for some real constant $M_{1}\geq 1$.

Under those assumptions, the spectrum of $P_{h}^{-1}A_{h}$ is positive and is contained in an interval determined by the constants $M_{0}$ and $M_{1}$ [21, Theorem 2.2], specifically, with $M_{01}:=1+\frac{1}{2M_{0}}-\frac{1}{2M_{1}}$,

(28) $$\frac{\lambda_{\max}(P_{h}^{-1}A_{h})}{\lambda_{\min}(P_{h}^{-1}A_{h})}\leq \frac{M_{1}+\sqrt{M_{1}^{2}-M_{1}}}{M_{01}-\sqrt{M_{01}^{2}-\frac{1}{M_{0}}}}\leq \frac{1}{2}{\left(1+\sqrt{5}\right)}^{2}M_{0}M_{1}.$$

Importantly, ${\overset{ˆ}{A}}_{h}\leq M_{0}A_{h}$ in the second condition is only required to hold on $\ker G_{h}=\Gamma_{h}$, where $A_{h}$ is positive definite by (22), and not on all of $X_{h}$.

We now specify our choice of ${\overset{ˆ}{A}}_{h}$ and ${\overset{ˆ}{S}}_{h}$. For ${\overset{ˆ}{A}}_{h}$ we take the block operator (25) with the first block replaced by

(29) $$2C_{\rho }\left({\left(\#T\right)}^{-2}{\left\|\partial_{t}\rho \right\|}_{H^{0,0}}^{2}+T^{-2}{\left\|\rho \right\|}_{H^{0,0}}^{2}\right),$$

while the second and the third nonzero blocks are multiplied by two. Here, $C_{\rho }>0$ is computed to satisfy(30) for all $f\in H^{1}(J)$. This entails ${\overset{ˆ}{A}}_{h}\geq 2A_{h}$, so that ${\overset{ˆ}{A}}_{h}>A_{h}$ is fulfilled in the first assumption. Up to normalization, (29) is the $H^{1,0}$ norm of *ρ*, and this being the space where *ρ* is sought, it is a natural positive definite regularization of the indefinite block of *A*. The normalization factors have been chosen with the motivation that the constant $C_{\rho }$ can be fixed independently of *T* and T, as long as the subintervals defined by T are of comparable length. Boundedness of ${\overset{ˆ}{A}}_{h}$ and $\Gamma_{h}$-ellipticity (22) of $A_{h}$ yield $\langle {\overset{ˆ}{A}}_{h}v,v\rangle \leq \left\|{\overset{ˆ}{A}}_{h}\right\|{\left\|v\right\|}_{X}^{2}\leq \left\|{\overset{ˆ}{A}}_{h}\right\|\alpha_{h}^{-1}\langle Av,v\rangle$ for any $v\in \ker G_{h}$, so that the second assumption is fulfilled with $M_{0}\leq \left\|{\overset{ˆ}{A}}_{h}\right\|\alpha_{h}^{-1}$. Alternatively, after discretization, if T is dense enough compared to the temporal discretization, the right hand side of (30) may be estimated by a multiple of . This multiple yields a possible value for $M_{0}\geq 2$, taking care of the first block in the relation ${\overset{ˆ}{A}}_{h}\leq M_{0}A_{h}$; the inequality holds trivially for the other two blocks.

To obtain ${\overset{ˆ}{S}}_{h}$ we exploit the fact that the composition $G_{h}{\overset{ˆ}{A}}_{h}^{-1}G_{h}^{\prime }$ is an isomorphism on $H^{0,0}$. Indeed, boundedness is clear, while positivity is obtained from using the inf-sup condition (23): $\langle G_{h}{\overset{ˆ}{A}}_{h}^{-1}G_{h}^{\prime }\lambda ,\lambda \rangle \geq {\left\|{\overset{ˆ}{A}}_{h}\right\|}^{-1}{\left\|G_{h}^{\prime }\lambda \right\|}_{X^{\prime }}^{2}\geq \beta_{h}^{2}{\left\|{\overset{ˆ}{A}}_{h}\right\|}^{-1}{\left\|\lambda \right\|}_{H^{0,0}}^{2}$. Therefore we take for ${\overset{ˆ}{S}}_{h}$ the $C_{\lambda }$-fold of the Riesz isomorphism on $H^{0,0}$, with, say, $0<C_{\lambda }<\beta_{h}^{2}{\left\|{\overset{ˆ}{A}}_{h}\right\|}^{-1}$. With this choice, the first assumption holds, and the constant $M_{1}$ in the third assumption may be taken as $\left\|{\overset{ˆ}{A}}_{h}^{-1}\right\|{\left\|G_{h}\right\|}^{2}$. In the numerical experiments below we use $C_{\lambda }:=\frac{1}{2}$.

It can be seen from the factorization (26) that the application of the inverse of the preconditioner only requires the application of ${\overset{ˆ}{A}}_{h}^{-1}$ and ${\overset{ˆ}{S}}_{h}^{-1}$ (as well as $G_{h}$ and $G_{h}^{\prime }$). The application of ${\overset{ˆ}{S}}_{h}^{-1}$ is trivial since we work with piecewise constant basis functions. In order to apply the block-diagonal operator ${\overset{ˆ}{A}}_{h}^{-1}$, it is useful to observe that due to the choice of space–time tensor product basis functions each block of ${\overset{ˆ}{A}}_{h}$ has the Kronecker product form T⊗X. The application of T⊗X and ${\left(T⊗X\right)}^{-1}=T^{-1}⊗X^{-1}$ is done by means of the matrix identity ${\left(T⊗X\right)}^{\pm 1}\mathrm{Vec}(u)=\mathrm{Vec}(X^{\pm 1}uT^{\pm T})$, where Vec stacks the columns of a matrix one after another into one long vector. In our Matlab implementation, we use this identity extensively and apply the direct solver to compute $X^{-1}u$ column-wise, $uT^{-T}$ row-wise, etc. This considerably speeds up the computation compared to the computation in the Vec form. Although we do not pursue this possibility here, we note that this tensor product structure allows to compute the unknown in a low rank tensor format (for instance the truncated singular value decomposition) [15,16] to reduce the memory requirements and the overall computational complexity. In the context of space–time discretization with PDE-constrained optimization this has been explored in [22].

## 5 Numerical examples

### 5.1 Robustness of the preconditioner

**Fig. 1:**
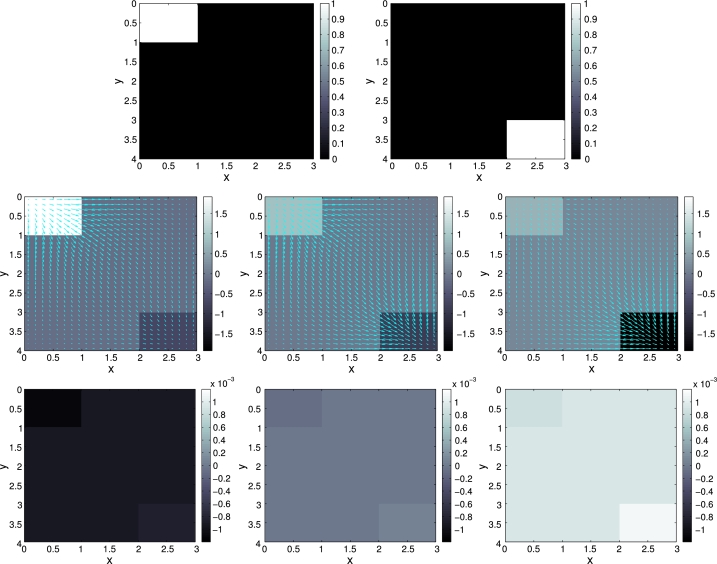
*Top*: Input data $\rho_{\tau }$ for *τ* = 0 (left) and *τ* = 2 (right). *Middle, left to right*: The divergence of the computed flux, ∇ ⋅ *j*, at *t* ≈ 0, *t* ≈ 1 and *t* ≈ 2. For the purpose of visualization, the flux is interpolated from the edges to the midpoint of each geometric element. *Bottom*: The computed source term *g* at *t* = 0,1,2. Note the small scale of $10^{-3}$ in accordance with the choice of $\alpha_{g}/\alpha_{j}=10^{4}$. See Section 5.1.

**Fig. 2:**
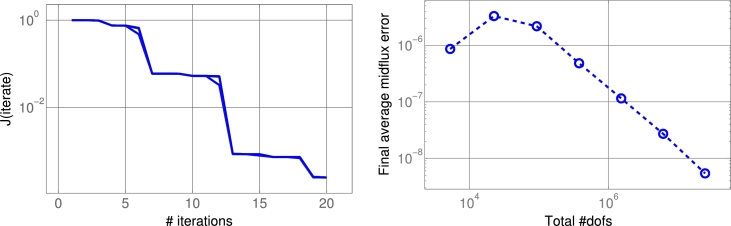
*Left*: The value of the cost functional J in the course of the conjugate gradient iteration. The curves for different spatial resolutions overlap. *Right*: First order convergence of the time-averaged flux over the horizontal midline as the spatial resolution is increased. See Section 5.1.

We investigate the robustness of the preconditioner with respect to the spatial discretization. The image domain is D:=(0,3)×(0,4) and the temporal interval is J:=(0,2). A sequence of partitions $D_{2^{-k}}$ for k=0,1,2,…,7, of *D* is obtained by subdividing *D* into $\left(3\times 2^{k})\times (4\times 2^{k}\right)$ equal rectangles. In each case, the partition $J_{h}$ of *J* consists of 100 subintervals of equal length; this choice is arbitrary but does not significantly affect the following results. The input data (2) consists of two frames $\rho_{0}$ and $\rho_{2}$, hence T={0,2}, as shown in Fig. 1. The parameter choices in the penalization functional (9) are $\alpha_{j}=10^{-4}$ and $\alpha_{g}=1$, which promotes the flux and puts a heavy penalty on the source term. We performed 20 CG iterations with the preconditioner (26) and the norm (27) as described in Section 4.2. The results are depicted in Fig. 1. In Fig. 2 (left) we report on the value of the cost functional in the course of the CG iteration, for different spatial resolutions levels *k*. We observe that the iteration is robust in *k*. In Fig. 2 (right), the convergence of the time-averaged flux over the horizontal midline (for the final CG iterate) as a function of the spatial resolution is shown. First order convergence in terms of the total number of degrees of freedom is observed.

To further test robustness of the preconditioner, we consider the energy error ${\left\|x^{⋆}-x_{i}\right\|}_{N_{h}P_{h}^{-1}A_{h}}^{2}$, where $x_{i}$ is the *i*-th CG iterate and $x^{⋆}$ is the exact solution (approximated by $x^{⋆}\approx x_{30}$). We compute the number of CG iterations that are necessary to reduce the initial energy error by a factor of 100. The maximum over the spatial discretization levels *k*, for different values of the regularization parameters $\alpha_{j}$ and $\alpha_{g}$, is shown in Table 1. At discretization level k=7, the system has close to 100*M* degrees of freedom. We see that this number does exhibit a dependence on the regularization parameters, but stays moderate and approximately constant across the considered range.

### 5.2 Vienna Prater movie

**Fig. 3:**
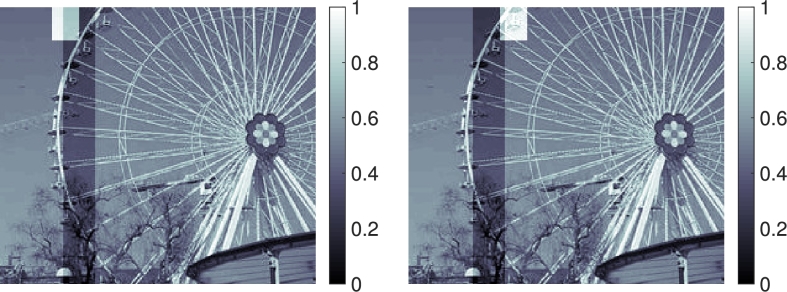
Two consecutive frames $\rho_{\tau }$ for *τ* ≈ 0.91 in the example in Section 5.2. Highlighted in white are the regions of [43,64]×[1,26] pixels (left) and [75,96]×[1,26] pixels (right). The Ferris wheel rotates clockwise and the superimposed dark vertical strip moves eastwards (→).

**Fig. 4:**
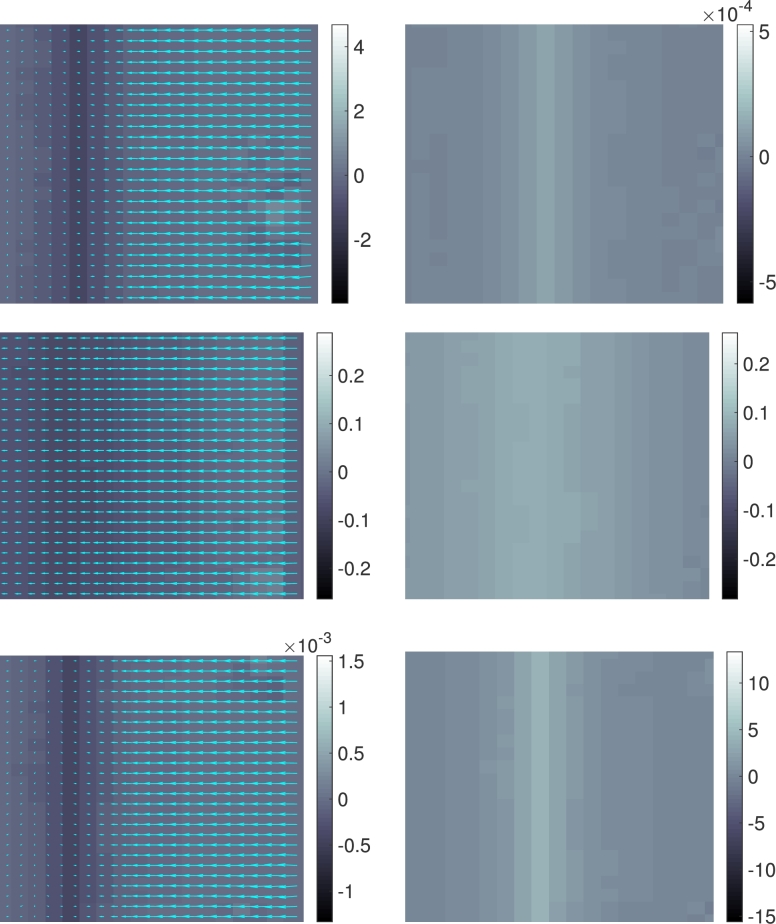
Computed discrete flux and source for the example in Section 5.2 in the region of [43,64]×[1,26] pixels. Top to bottom, the values of $\left(\alpha_{j},\alpha_{g}\right)$ are $\left(10^{-4},1\right)$, $\left(10^{-2},10^{-2}\right)$, and $\left(1,10^{-4}\right)$. Left: The computed discrete flux *j* with the background color showing the divergence ∇ ⋅ *j*. Right: The computed source *g*.

**Fig. 5:**
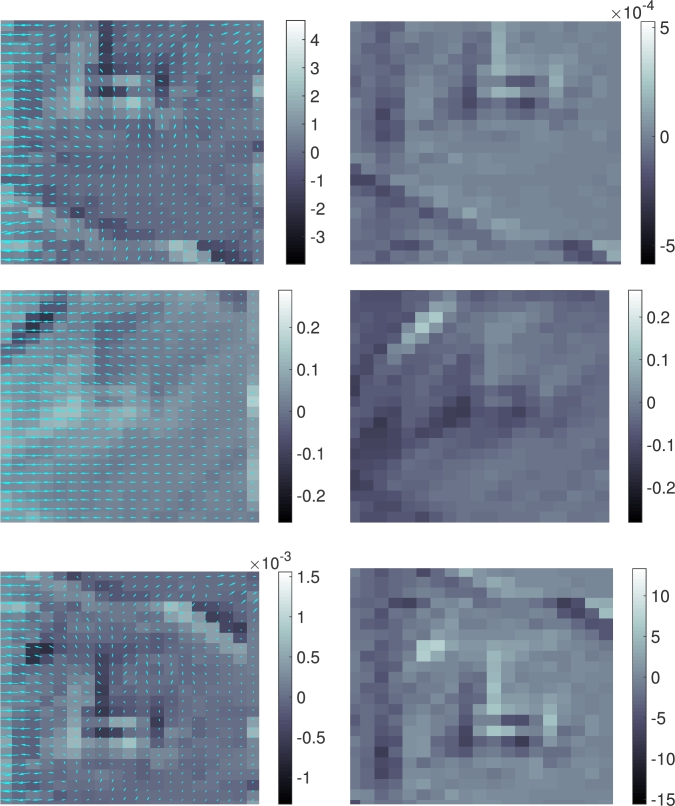
Computed discrete flux and source for the example in Section 5.2 in the region of [75,96]×[1,26] pixels. Top to bottom, the values of $\left(\alpha_{j},\alpha_{g}\right)$ are $\left(10^{-4},1\right)$, $\left(10^{-2},10^{-2}\right)$, and $\left(1,10^{-4}\right)$. Left: The computed discrete flux *j* with the background color showing the divergence ∇ ⋅ *j*. Right: The computed source *g*.

In this example we apply the method to a movie showing the Ferris wheel at the Vienna Prater park turning clockwise. A vertical dark strip moving from left to right across the image has been superimposed artificially, simulating shading, see Fig. 3. The movie consists of 200 frames of 256×216 pixels each. This data is mapped to the domain D=(0,4)×(0,3) and the temporal interval J=(0,2). We look at frames 91 and 92 and zoom in on two regions highlighted in Fig. 3. The computed discrete flux between these two frames is shown in Fig. 4 for the first region, and in Fig. 5 for the second region, for different values of the regularization parameters. In both cases, there is a strong flux westward (←) across the superimposed dark strip, which moves eastwards (→). There is a less distinct flux directed north–east (↗) across the bright spokes of the Ferris wheel (in the second region). It is interesting to note that the model generates a strong, almost uniform flux *j* across the superimposed dark strip in the opposite direction of the movement of the strip in order to transport the bright intensity from its bow to its stern. One observes finer flow pattern within the strip away from its boundary caused by the rotation of the Ferris wheel, as the graycolor displaying ∇⋅j in Fig. 5 suggests. On the other hand, the moving bright spokes of the Ferris wheel cause the intensities to flow across them in the same direction (as one might expect from the optical flow model). Concerning the role of the regularization parameters $\alpha_{j}$ and $\alpha_{g}$, we observe in Fig. 5 meaningful results for $\left(\alpha_{j},\alpha_{g})=(10^{-4},1\right)$ and $\left(\alpha_{j},\alpha_{g})=(1,10^{-4}\right)$, which are, up to scaling, visually very similar. This is so because, roughly speaking, $\partial_{t}\rho$ is decomposed into two parts that relate to each other as $1:10^{-4}$, and each part is captured by either ∇⋅j or *g*. For $\alpha_{j}=\alpha_{g}=10^{-2}$ we observe qualitatively different and less meaningful results, presumably because of overpenalization in the functional (10).

## 6 Conclusions

We have considered a version of the optical flow equations in which the image brightness evolves as a conserved quantity up to possible sources (or sinks). We have formulated the problem of estimating the optical flow and the source as an energy minimization problem. We have investigated a space–time discretization and preconditioning strategy for the resulting saddle-point equations. The discretization was shown to be stable in the Galerkin sense, and the preconditioner to be robust in the discretization resolution albeit with a mild dependence on the regularization parameters. The conjugate gradient method (with a suitable scalar product for which the preconditioned system matrix is symmetric and positive definite) allows to solve the complete space–time problem within a few iterations. The transport equation model with the chosen penalization functional produces rather global flows; we do not expect this to happen if the “kinetic energy” ${\left\|j\right\|}^{2}/\rho$ is penalized instead of the divergence of the flux *j* and wish to investigate this type of penalization next. We note, however, that our model is typical for control problems with a quadratic cost functional subject to a linear time-dependent PDE constraint, and we therefore expect the results to be applicable elsewhere.

## Figures and Tables

**Table 1:** Number of CG iterations to reduce the initial energy error by a factor of 100 for various choices of the regularization parameters $\alpha_{j}$ (horizontal) and $\alpha_{g}$ (vertical). The number shown is the maximum over the spatial discretization levels *k* = 0,…,7. See Section 5.1.

$\alpha_{g}$	$\alpha_{j}$				
$10^{-4}$	$10^{-3}$	$10^{-2}$	$10^{-1}$	10^0^
10^2^	13	11	10	9	5
10^1^	13	11	10	8	5
10^0^	13	11	10	8	5
$10^{-1}$	13	11	9	8	7
$10^{-2}$	12	10	8	6	5
